# Complete Genome of a Panresistant *Pseudomonas aeruginosa* Strain, Isolated from a Patient with Respiratory Failure in a Canadian Community Hospital

**DOI:** 10.1128/genomeA.00458-17

**Published:** 2017-06-01

**Authors:** Jianhui Xiong, Maxime Déraspe, Naeem Iqbal, Sigmund Krajden, William Chapman, Ken Dewar, Paul H. Roy

**Affiliations:** a Department of Laboratory Medicine, St. Joseph’s Health Centre, Toronto, Ontario, Canada; b Division of Infectious Diseases, St. Joseph’s Health Centre, Toronto, Ontario, Canada; c Department of Laboratory Medicine and Pathobiology, University of Toronto, Ontario, Canada; d Centre de Recherche en Infectiologie, and Department de Biochimie, de Microbiologie, et de Bio-informatique, Université Laval, Québec City, Québec, Canada; e McGill University and Genome Québec Innovation Centre, Montreal, Québec, Canada

## Abstract

We report here the complete genome sequence of a panresistant *Pseudomonas aeruginosa* strain, isolated from a patient with respiratory failure in Canada. No carbapenemase genes were identified. Carbapenem resistance is attributable to a frameshift in the *oprD* gene; the basis for colistin resistance remains undetermined.

## GENOME ANNOUNCEMENT

Early in 2014, a *Pseudomonas aeruginosa* strain (E6130952) was isolated from the induced sputum of a patient with respiratory failure. It was resistant to all the tested antibiotics using both Vitek 2 (Montreal BioMérieux, Montreal, Canada) and disk-diffusion methods, and panresistance was confirmed by the agar dilution method in a reference laboratory. The tested antibiotics included ceftazidime, piperacillin-tazobactam, carbapenems, gentamicin, amikacin, ciprofloxacin, and colistin. The patient had repeated acute exacerbations of chronic respiratory infections with *P. aeruginosa*, including an episode of bacteremia, and failed to survive. Previous isolates of *P. aeruginosa* from the same patient were not fully resistant to ceftazidime and carbapenems, and no colistin testing was performed. Unfortunately, the precursor strains were not preserved for comparison in this study. The patient had no history of travel to South or East Asia and had undergone treatments with broad-spectrum antibiotics, but no colistin treatment was noted.

The genome was sequenced by the single-molecule real time technique using the Pacific Biosciences RSII platform (Pacific Biosciences, Menlo Park, CA, USA) at the McGill University Genome and Québec Innovation Centre. The genome was assembled *de novo* using the Hierarchical Genome Assembly Process (HGAP) (1). Further editing and manual annotation were carried out using RAST (2) and Artemis version 13.2.0 (3).

Genome analysis revealed a chromosome with a length of 7,040,952 bp and a single plasmid with a length of 36,454 bp. The plasmid did not contain any known antibiotic resistance genes. Analysis of the genome revealed no acquired carbapenemase or *mcr-*1 genes, but it did contain a frameshift in the outer membrane *oprD* gene, which confers carbapenem resistance.

The strain is genetically most similar to *P. aeruginosa* NCGM1984 (AP014646) and NCGM1900 (AP104622), which harbor two copies of the carbapenem resistance gene *bla*_IMP-34_ and were isolated in Japan (4), and to NCGM2.S1 (AP012280), which harbors one copy of *bla*_IMP-1_ (5). Most of the contents of the genomic islands are shared with the first two of these strains. The PmrAB, PhoPQ, and lipid A-Ara4N pathways, which may contribute to colistin resistance, were identified, as well as various RND, MFS, MATE, and ABC family MDR efflux pumps, such as MexCD-OprJ and MexEF-OprN. The *pmrAB* and *phoPQ* genes contain the same seven mutations found in some strains in the study by Lee and Ko (6). Paradoxically, the *phoPQ* genes of NCGM1984 and NCGM1900 are disrupted by one of the *bla*_IMP-34_-containing integrons, but these strains are sensitive to colistin. Genomic islands were identified by comparison to the sequence of *P. aeruginosa* PAO1 and other strains (7). We identified two class 1 integrons: *intI1-aacA8-oxaA2-aacA7-gcuD-qacEdelta1-sul1-orf5-tniBdelta1-tniA* and *intI1-aadB-qacEdelta1-sul1-orf5*. Apart from the acquisition of *mcr-1*, other mechanisms of colistin resistance remain unclear, although multiple processes that involve mutations in transcriptional regulators and colistin-dependent expression have been reported (8–10).

Panresistant *P. aeruginosa* strains are a major threat to the therapeutic management of patients infected with this organism. Multiple resistance mechanisms are involved in the evolution of panresistance and further studies and surveillance of similar organisms are warranted in order to find a better clinical solution to this health threat.

### Accession number(s).

The chromosome and plasmid sequences were deposited in GenBank under the accession numbers CP020603 and CP020602, respectively.
